# Chronic Diseases, Health Behaviors, and Demographic Characteristics as Predictors of Ill Health Retirement: Findings from the Korea Health Panel Survey (2008–2012)

**DOI:** 10.1371/journal.pone.0166921

**Published:** 2016-12-08

**Authors:** Young Joong Kang, Mo-Yeol Kang

**Affiliations:** 1 Occupational Safety and Health Research Institute, Korea Occupational Safety and Health Agency, Ulsan, South Korea; 2 Department of Preventive Medicine, College of Medicine, Seoul National University, Seoul, South Korea; Cardiff University, UNITED KINGDOM

## Abstract

**Purpose:**

The research aim was to identify demographic characteristics, chronic diseases, and unhealthy behaviors predicting ill health retirement in South Korea.

**Methods:**

Data were collected from 15,407 individuals enrolled in the first through the fifth phases of the Korea Health Panel Survey (2008–2012) using structured questionnaires examining retirement, morbidities, and health-related behaviors. The Cox proportional hazard model was used to examine demographic and clinical characteristics’ effects on ill health retirement. Lost years of working life expectancy were calculated for demographic and clinical characteristics.

**Results:**

Older, female, and manual workers were more likely to experience ill health retirement, as were respondents reporting poor health-related habits (e.g., heavy drinking, irregular meals, less sleep hours, obesity, and no regular exercise). The chronic diseases most closely associated with ill health retirement were, in order, psychiatric disease, ophthalmologic disease, neurologic disease, infectious disease, and musculoskeletal diseases. The average reduction in working life expectancy was 9.73 years.

**Conclusions:**

Our study results can help contribute to the development of strategies for reducing the risk of ill health retirement and promoting sustainable labor force participation in an aging society.

## Introduction

An aging population is an important challenge confronted by most industrial countries [1]. While life expectancy has increased with the improvement of health status and overall living conditions in most developed Western countries, older workers retire earlier than before [2]. This tendency is rarely sustainable due to financial pressures and is incompatible with labor deficiencies in an aging society. Therefore, it is necessary to understand the ageing of the workforce and the role of health status in continuing employment.

The relationship between unemployment and health is quite strong, and considerable research has been published on this issue. In the past, several longitudinal studies have demonstrated the adverse effects of unemployment on poor health. Early retirement has become a social issue related to public health and social welfare in a bid to balance the timing between early labor force exits and the pension budget. Therefore, it is important to identify specific factors affecting early retirement in order to reduce the risk of early retirement.

Epidemiological research has emerged regarding the reverse direction of causality possible in the relationship between unemployment and health; poor health and poor health habits may lead to unemployment and early retirement [3]. In an 11-year follow up study of Finnish workers, poor health predicted early retirement through both ill health-based and non-ill-health-based early pension schemes [4]. Likewise, in an Australian study, individuals aged 45–64 years with chronic ill health conditions were significantly more likely not to be in the labor force compared to those without chronic health conditions. The authors estimated that those aged 45–64 years who are out of the labor force reduce their national GDP by approximately $14.7 billion per annum [5].

Adding to this significant exiting of labor participation by older workers, Korea has distinctive characteristics that cause a greater need to be concerned about early retirement. Korea is distinguished by low unemployment rates among those aged over 55 years. In 2013, unemployment rates were 2.1% for those aged 55 to 64 years, and 1.5% for those over 65 years. These rates are lower than the mean levels reported for the Organization for Economic Co-operation and Development (OECD) countries (5.6% [55 to 64 years], and 3.1% [≥.65 years] respectively) [6]. This difference might be caused by the low guaranteed Korean social security service system. The basic old-age pension coverage rate only accounted for 28% of all Korea elderly people [7]. In this context, unemployment and early retirement can be more desperate and threatening to health states.

Korea, as well as Japan, will need to confront a rapidly changing population structure [1]. Thus, early retirement is an important socio-economic and public health-related issue, especially in East Asia. However, few studies have examined ill health retirement (IHR) in East Asia, in contrast with Western countries [5, 8, 9]. Hence, it is necessary to be fully aware of the emerging welfare problem in an Asian aging society. The aim of this research is to demonstrate which demographic characteristics, chronic diseases, and unhealthy behaviors can predict IHR by analyzing a large longitudinal sample of representative data from the Korean population. This analysis provides insight into how to cope with early retirement-related issues in aging societies.

## Materials and Methods

### Data collection and participants

Data were collected from a sample from the first through to fifth waves of the Korea Health Panel Survey (KHPS), which was conducted by the Korea Institute for Health and Social Affairs and the National Health Insurance Corporation to produce representative and reliable statistics about the use of health services and health expenditures on a national scale. The samples were extracted from 90% complete enumeration data of the 2005 Population and Housing Census. Sampling units were households selected through a stratified, multistage, probability sampling design based on geographic area using household registries (e.g., 16 cities, provinces, *dongs*, *eups*, *myeons*, and *gus*). Surveys were conducted in 2008, 2009, 2010, 2011, and 2012. The KHPS collected data for a total of about 8,000 households, with a response rate of 94.1%,;examined 7,201 households and 22,595 respondents who are non-institutionalized Korean adults populations 20 year old or more at Wave 1. Households selected for the Korea Health Panel sample were interviewed once or twice per year. The second survey in 2009 was a follow-up with 19,153 subjects, representing 84.8% of the original sample; the third survey in 2010 was administered to 17,885 subjects, 79.2% of the original sample; the fourth survey in 2011 was administered to 17,035 subjects, 75.4% of the original sample; and, the fifth survey in 2012 was administered to 15,872 subjects, 70.2% of the original sample.

The KHPS is a national public database (https://www.khp.re.kr) that includes an identification number for each household and each member; however, the number is not associated with any personal identifying information, and the data collection system and database were designed to protect respondent confidentiality. Respondents were required to read and sign an agreement form before participating in the KHPS and to consent that their data could be used in future scientific research.

The survey comprised about 200 questions. Questionnaire interviews were conducted by trained interviewers. The survey was divided into the following categories: household-based, individual-based, and disease-based studies. The household study included questions about general characteristics, living expenses, purchase of pharmaceutical products, and private health insurance and household premiums. The individual study considered the demographic characteristics of the respondents. The disease-based study included those with chronic diseases, inpatients, outpatients, and emergency service use. For chronic disease, survey respondents were asked whether they had a chronic disease; if they responded positively, they were asked whether they had been diagnosed by a physician.

**Fig 1:**
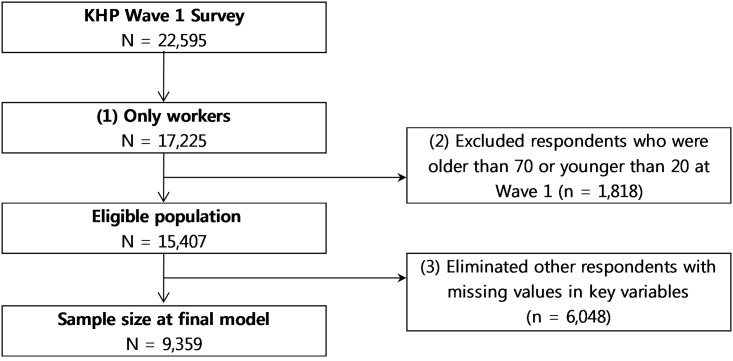
Schematic diagram depicting the study population.

The inclusion/exclusion criteria were as follows: (1) during the first phase, workers (*n* = 17,225) were selected from the total sample (*N* = 22,595), and (2) we restricted our sample to individuals aged 20–70 years (*n* = 15,407). (3) We also excluded subjects without data on health behaviors (n = 3,333) at wave 2 or on employment status (n = 2,182) at wave 4. After further excluding observations with missing values, the final sample for analysis comprised 9,359 adults. The survey related to health behaviors was performed only from Wave 2 (2009), so that the eligible population for analysis of health behaviors was 14,509 (Fig 1).

### Study variables and measurements

New cases of IHR were defined as those who retired due to their health problems before their scheduled or regular retirement age in one of the follow-up surveys. Moreover, worker who died from disease before retirement were also included as an event of IHR. Cases of retirement due to other reasons (e.g., becoming a carer for a family member) were not considered as events, but as censored data. The follow-up period was calculated as the difference between the date of the first survey and the date of the survey-identified IHR. We organized the respondents in ascending order of time for the follow-up. If a respondent had more than one retirement event during the study period, we chose the first event for the calculation of the follow-up period. If working respondents were lost to follow-up across the second to fourth waves of the surveys, they were all regarded as censored data and their follow-up period was calculated as the difference between the date of the first survey and the date of the final survey that they completed. For the remainder of the respondents who did not experience an event or were not lost to follow up, the follow-up period was calculated as the difference between the date of the first survey and the last date of the fourth survey.

The KHPS uses 298 diagnostic codes that were developed based on the disease classification systems of Western and traditional Korean medicine. We classified these into the following categories: infectious disease, cancer, hematologic disease, psychiatric disease, neurologic disease, ophthalmologic disease, otologic disease, circulatory disease, respiratory disease, digestive disease, endocrine disease, dermatologic disease, musculoskeletal disease, urologic disease, injury, and others.

Information on health behaviors (e.g., smoking habits, alcohol consumption, regular exercise, body mass index; BMI) were collected during the second phase of the survey in 2009. Physical activity was categorized as regularly performed or not regularly performed. Regular exercise was defined as exercise more than twice per week with each session lasting at least 30 minutes. Smoking behavior was categorized as current, past, and non-smoker. We defined heavy drinking as consuming at least 7 glasses of alcohol in a single occasion for males and 5 glasses for females for at least 8 days in the past 30 days. BMI was calculated using height and weight variables as weight/height^2^ (kg/m^2^). BMIs ≧25.0 were regarded as obese, and BMIs of 23–25 were regarded as overweight, following the World Health Organization Asia-Pacific guidelines [10].

Occupation was categorized into the following groups: managers and professionals, office workers, service and sales workers, agriculture, forestry, and fisheries workers, craft and device machine operators, assembly workers, manual workers, and others.

### Statistical analysis

We listed the frequencies of the respondents’ baseline characteristics and compared them to each categorized variable to analyze the respondents’ general characteristics. To explore whether diagnosed diseases, lifestyle behaviors, and demographic characteristics affect IHR, we analyzed the distribution of each group using chi-square tests and analysis of variance.

Cox proportional hazard models were used to evaluate the effects of diagnosed diseases, lifestyle behaviors, and demographic characteristics on IHR. The models were comprised of Model 1 (crude), Model 2 (adjusted for age and gender), and Model 3 (adjusted for all variables). Covariates associated with events were determined using stepwise Cox regression analysis. Two approaches were used to assess the validity of the proportional hazards assumption. First, we examined graphs of the log-minus-log-survival functions and found that the plots had grossly parallel lines. Second, we used time-dependent covariates to confirm proportionality and found that none of the time-dependent covariates were statistically significant, suggesting that the hazard is reasonably constant over time.

Working life expectancy represents the number of expected future years in the workforce; therefore, lost years of working life expectancy comprised the number of lost years due to IHR before the expected retirement age in Korea (65 years). The proportion of IHR before age 65 and lost years of working life expectancy was assessed using demographic and clinical characteristics.

Statistical analysis was performed using SAS (Version 9.3, SAS Institute, Cary, NC, USA). A two-tailed p < 0.05 was considered significant.

## Results

Respondents’ mean age was 48.1 ± 11.4 years; 54.71% were male. Approximately 40% had no diagnosed disease; the most frequently diagnosed disease was musculoskeletal disease (11.72%). The proportion of heavy drinkers was 13.79%; 94.20% of these were male. Over a quarter were obese and almost half of respondents did not exercise regularly. About 16% of respondents slept less than 6 hours per day. Table 1 presents results from the preliminary analysis of the frequency of health-related early retirement according to each variable.

**Table 1:** General characteristics of employed individuals at baseline.

	Distribution		Health-related early retirement^a^	
**Characteristics**	**n**	**%**	n	%
**Demographics**				
Gender				
Male	8429	54.71	429/6692	6.40
Female	6978	45.29	420/4743	8.86
Age				
20–40	3039	19.72	22/2035	1.08
40–50	3858	25.04	118/3069	3.84
50–60	4475	29.05	229/3427	6.96
60–70	4035	26.19	470/2904	16.12
Occupation				
Managers and professionals	2789	18.10	51/2086	2.44
Office workers	1288	8.36	27/923	2.93
Service and sales workers	3240	21.03	162/2329	6.94
Agriculture, forestry, and fisheries workers	2172	14.10	156/1840	8.46
Craft and device machine operators, and assembly workers	3147	20.43	97/2474	3.92
Manual workers	2720	17.65	349/1746	19.92
Others	51	0.33	7/37	18.92
**Chronic Diseases**				
No	6336	41.12	89/4645	1.92
Infectious disease	306	1.99	37/244	14.86
Cancer	275	1.78	24/188	12.70
Haematologic disease	61	0.40	1/39	2.56
Psychiatric disease	146	0.95	35/107	32.71
Neurologic disease	170	1.10	19/125	15.20
Ophthalmologic disease	328	2.13	52/242	21.40
Otologic disease	96	0.62	4/72	5.56
Circulatory disease	1810	11.75	169/1307	12.91
Respiratory disease	546	3.54	10/410	2.44
Digestive disease	1621	10.52	133/1254	10.60
Endocrinologic disease	882	5.72	61/653	9.33
Dermatologic disease	412	2.67	13/309	4.21
Musculoskeletal disease	1817	11.79	172/1393	12.32
Urologic disease	393	2.55	14/291	4.81
Injury	111	0.72	6/86	6.98
Others	97	0.63	10/70	14.29
**Total**	15,407		849/11435	7.41
	Distribution		Health-related early retirement^b^	
**Health behaviors at Wave 2 (n = 14,509)**	**n**	**%**	n	%
Smoking (missing = 517)				
Non-smoker	7766	55.50	351/5751	6.10
Ex-smoker	2419	17.29	90/1946	4.61
Current smoker	3807	27.21	223/2982	7.48
Heavy drinking (missing = 308)				
No	12242	86.21	586/9224	6.35
Yes	1959	13.79	83/1611	5.15
Regular exercise (missing = 310)				
No	6293	44.32	311/4765	6.53
Yes	7906	55.68	359/6069	5.89
Sleeping hours (missing = 4612)				
<6 hours	2188	16.83	183/1676	10.92
≥6 hours	10812	83.17	462/8221	5.62
BMI (missing = 346)				
Normal or underweight	6184	43.66	301/4601	6.53
Overweight	3883	27.42	152/3041	5.00
Obese	4096	28.92	216/6.68	6.81
**Total**	14,509		681/11054	6.16

^a^Excluded retirees due to business closure, layoff, or family problems other than health problems (n = 3972).

^b^Excluded retirees due to business closure, layoff, or family problems other than health problems (n = 3455).

Female, older, and manual workers were more likely to experience IHR. Additionally, IHR was frequent among those with the following conditions, in order: psychiatric disease, ophthalmologic disease, neurologic disease, infectious disease, and musculoskeletal disease. Specific diseases and conditions related to unemployment during our observation are listed in S1 Table. Moreover, IHR was more common in respondents who had poor health-related habits (e.g., heavy drinking, irregular meals, fewer sleep hours, obesity, no regular exercise). The remaining descriptive characteristics of the study population are listed in Table 1.

Table 2 presents hazard ratios (HRs) of health-related early retirement according to demographic and clinical characteristics, exhibiting a pattern similar to the results in Table 1. Individuals with infectious disease, psychiatric disease, neurologic disease, ophthalmologic disease, circulatory disease, respiratory disease, digestive disease, dermatologic disease, musculoskeletal disease, and urologic disease had a significantly higher risk of health-related early retirement after adjusting for all variables. The risk of IHR was significantly greater among respondents who were current smokers, did not exercise regularly, slept less than 6 hours, and were obese. Service and sales workers had a 1.78-fold increased risk for IHR, and manual workers had the highest risk compared to respondents in other occupations (HR = 3.33).

**Table 2:** Health and behavioral factors influencing ill health retirement in total respondents.

	Model 1			Model 2			Model 3		
	HR	95% CI		HR	95% CI		HR	95% CI	
**Chronic Diseases**									
No	1	reference		1	reference		1	reference	
Infectious disease	7.339	5.002	10.768	4.629	3.140	6.825	5.000	3.302	7.573
Cancer	6.534	4.163	10.256	4.762	3.140	6.825	2.394	1.303	4.397
Haematologic disease	1.225	0.171	8.789	0.875	0.122	6.288	0.927	0.129	6.676
Psychiatric disease	17.796	12.036	26.312	11.077	7.443	16.486	5.800	3.743	8.986
Neurologic disease	7.588	4.624	12.452	5.880	3.387	9.192	3.745	2.082	6.735
Ophthalmologic disease	11.239	7.982	15.823	5.944	4.174	8.466	3.695	2.462	5.545
Otologic disease	2.869	1.054	7.814	1.519	0.556	4.151	1.378	0.504	3.771
Circulatory disease	6.563	5.077	8.484	3.511	2.684	4.592	2.600	1.956	3.456
Respiratory disease	1.219	0.634	2.344	0.924	0.480	1.780	1.354	0.768	2.387
Digestive disease	5.362	4.100	7.013	3.373	2.561	4.443	2.645	1.985	3.524
Endocrine disease	4.633	3.345	6.417	2.668	1.913	3.723	1.655	0.903	3.032
Dermatologic disease	2.062	1.152	3.690	1.764	0.985	3.158	2.231	1.661	2.997
Musculoskeletal disease	6.147	4.759	7.940	3.185	2.426	4.181	2.074	1.241	3.464
Urologic disease	2.455	1.397	4.313	1.439	0.816	2.539	2.506	1.245	5.042
Injury	3.618	1.583	8.270	2.132	0.930	4.889	1.236	0.389	3.925
Others	7.523	3.913	15.464	3.424	1.766	6.637	2.351	1.658	3.333
**Health behaviors at Wave 2**									
Smoking									
Non-smoker	1	reference		1	reference		1	reference	
Ex-smoker	0.747	0.592	0.941	1.092	0.808	1.477	1.014	0.750	1.373
Current smoker	1.234	1.043	1.460	2.317	1.810	2.966	2.268	1.759	926
Heavy drinking									
No	1	reference		1	reference		1	reference	
Yes	0.815	0.647	1.025	0.988	0.775	1.261	1.007	0.786	1.289
Regular exercise									
No	1.126	0.967	1.310	1.318	1.132	1.536	1.383	1.182	1.617
Yes	1	reference		1	reference		1	reference	
Sleeping hours									
<6 hours	1.952	1.6455	2.316	1.672	1.407	1.987	1.483	1.245	1.768
≥6 hours	1	reference		1	reference		1	reference	
BMI									
Normal or underweight	1	reference		1	reference		1	reference	
Overweight	0.748	0.615	0.909	0.634	0.521	0.771	0.728	0.598	0.888
Obese	1.028	0.864	1.225	0.935	0.784	1.113	0.994	0.828	1.193
**Occupation**									
Managers and professionals	1	reference		1	reference		1	reference	
Office workers	0.753	0.414	1.370	0.946	0.520	1.723	0.800	0.439	1.461
Service and sales workers	2.786	2.000	3.881	1.954	1.388	2.751	1.781	1.261	2.516
Agriculture, forestry, and fisheries workers	2.518	1.782	3.560	1.045	0.724	1.508	1.014	0.697	1.476
Craft and device machine operators, and assembly workers	1.589	1.110	2.275	1.144	0.797	1.642	1.096	0.762	1.577
Manual workers	7.507	5.498	10.251	3.900	2.812	5.409	3.330	2.388	4.645

Model 1 is not adjusted.

Model 2 is adjusted for age and gender.

Model 3 is adjusted for all variables.

Stepwise Cox regression analysis determined the models best fitting the data. Gender, age, smoking, regular exercise, BMI group, sleeping hours, chronic diseases, and occupation significantly predicted IHR (df = 30, p < 0.0001; Table 3). The likelihood ratio chi-square statistic was used to compare the fitted model to a model without covariates, and the -2 log likelihood statistic showed the overall significance of the set of covariates included in the final model (p < 0.0001).

**Table 3:** Results of stepwise selection for predictive factors of ill health retirement.

	HR	95% CI	
**Age**	1.057	1.046	1.068
**Smoking**			
Non-smoker	1	reference	
Ex-smoker	0.864	0.679	1.100
Current smoker	1.936	1.621	2.313
**Regular exercise**			
No	1.385	1.184	1.620
Yes	1	reference	
**BMI**			
Normal or underweight	1	reference	
Overweight	0.727	0.597	0.887
Obese	0.997	0.831	1.197
**Sleeping hours**			
<6 hours	1.500	1.260	1.786
≥6 hours	1	reference	
**Chronic diseases**			
No	1	reference	
Infectious disease	4.883	3.229	7.385
Cancer	2.434	1.326	4.468
Haematologic disease	0.943	0.131	6.790
Psychiatric disease	5.841	3.770	9.052
Neurologic disease	3.801	2.115	6.833
Ophthalmologic disease	3.774	2.517	5.658
Otologic disease	1.404	0.513	3.840
Circulatory disease	2.592	1.950	3.445
Respiratory disease	1.347	0.764	373
Digestive disease	2.658	1.996	3.540
Endocrine disease	1.650	0.901	3.022
Dermatologic disease	2.282	1.701	3.061
Musculoskeletal disease	2.102	1.259	3.510
Urologic disease	2.588	1.287	5.201
Injury	1.207	0.380	3.829
Others	2.336	1.648	3.311
**Occupation**			
Managers and professionals	1	reference	
Office workers	0.816	0.447	1.488
Service and sales workers	1.885	1.343	2.646
Agriculture, forestry, and fisheries workers	1.057	0.729	1.532
Craft and device machine operators, and assembly workers	1.096	0.762	1.577
Manual workers	3.466	2.494	4.817

Table 4 shows the comparison of the proportion of IHR before 65 years of age and lost years of working life expectancy according to the demographic and clinical characteristics of Korean adults. The results showed that 72.2% of IHR occurred before the expected retirement age in Korea (65 years) and the average reduction in working life expectancy was 9.73 years. The lost years of working life expectancy of people with psychiatric disease and office workers were notably greater than that of the general population.

**Table 4:** IHR^a^ before age 65 and lost years of working life expectancy.

Characteristics	IHR total	IHR before age 65		Lost years of working life expectancy due to IHR	
		*n*	%	Mean	*SD*
**Demographics**					
Gender					
Male	429	308	71.79	10.39	7.89
Female	420	305	72.62	9.06	7.04
Occupation					
Managers and professionals	51	51	100.00	11.59	7.52
Office workers	27	17	62.96	17.82	11.91
Service and sales workers	162	144	88.89	9.25	5.96
Agriculture, forestry, and fisheries workers	156	73	46.79	6.05	5.94
Craft and device machine operators, and assembly workers	97	69	71.13	12.25	8.17
Manual workers	349	259	74.21	9.46	7.49
**Chronic diseases**					
Infectious disease	37	26	70.27	9.81	7.06
Cancer	24	24	100.00	9.95	5.70
Haematologic disease	1	0	0.00	.	.
Psychiatric disease	35	30	85.71	19.63	7.05
Neurologic disease	19	16	84.21	8.38	6.97
Ophthalmologic disease	52	46	88.46	5.48	3.85
Otologic disease	4	3	75.00	3.67	2.31
Circulatory disease	169	107	63.31	7.82	5.47
Respiratory disease	10	7	70.00	3.71	4.72
Digestive disease	133	87	65.41	10.09	7.35
Endocrine disease	61	50	81.97	7.80	5.35
Dermatologic disease	13	11	84.62	7.27	1.35
Musculoskeletal disease	172	111	64.53	8.02	5.69
Urologic disease	14	11	78.57	12.82	6.21
Injury	6	3	50.00	10.00	7.81
Others	10	4	40.00	9.25	9.95
**Health behaviors at Wave 2**					
Smoking					
Ex-smoker	88	38	43.18	10.45	6.82
Current smoker	194	151	77.84	9.68	7.93
Heavy drinking	78	62	79.49	12.66	8.88
Lack of exercise	321	250	77.88	10.01	8.37
Lack of sleep (<6 hours/day)	393	265	67.43	9.21	7.92
BMI					
Overweight	135	122	90.37	9.37	6.83
Obese	191	129	67.54	9.96	8.73
**Total**	849	613	72.20	9.73	7.51

^a^IHR: ill health retirement

## Discussion

The current results indicated that older, female, and manual workers were more likely to experience ill health retirement; early retirement was also more common among respondents with poor health-related habits (e.g., heavy drinking, irregular meal, less sleep hours, obesity, no regular exercise). Chronic diseases (e.g., psychiatric disease, ophthalmologic disease, neurologic disease, infectious disease, and musculoskeletal disease) increased the risk of ill health retirement.

Wide variations in the rates of early retirement have been reported between employees. Some variation is to be expected (e.g., a higher rate in jobs that require high levels of physical fitness or an older workforce [11]). Our results additionally suggest that older and manual workers are more likely to experience IHR. Another study finding that female workers have a 1.34-fold increased risk of IHR can be partially explained by the effect of non-medical factors on decisions to retire. It is reasonable to assume that women are more likely to exit the labor market than men in response to familial expectations [12] and that their retirement is strongly affected by their husbands’ retirement decisions [13, 14].

Life expectancy increased with the improvement of health status and as an individual’s overall living condition developed; however, older workers exit the labor market in great numbers in most developed countries. Accordingly, the fear is that this will necessitate high tax rates for the current, shrinking workforce. An ageing population may lead to a shortage of workers and an increased dependency ratio: as older workers leave the labor market earlier and the share of younger aged workers in the labor force declines [2, 15], more people may claim pension benefits with fewer people working and paying income taxes. This combination of higher spending commitments and a smaller tax-paying population is a source of concern for the governments of developed countries. In aging populations and societies with pension crises, promoting workforce participation rates among the elderly is key to alleviating the negative effects of an aging work force.

The OECD suggests that encouraging female labor participation is key to managing the effect of an aging labor force in South Korea. They projected that if the female participation rate in Korea were to increase to the current level for males for each age group by 2050, the total labor force would only decline to around 25.6 million, which is almost 19% higher than if the participation rates were to unchanged [16]. In a study regarding the implications of population aging for economic growth in OECD countries, it was found that female labor participation could diminish the aging labor force impact, taking into account female labor force participation changes and the predicted labor force in relation to population and economic growth changes for 2050 [17]. The estimated results suggested that greater female labor force participation can mitigate the economic consequences of an ageing society. Another study, which analyzed the Korean elderly population using the Korean Longitudinal Study of Ageing (KLoSA) dataset (2006–2012), found that smoking, obesity, hypertension, malignancy, cardiovascular disease, and arthritis are major determinants of IHR among female workers [18]. Prevention and management of those conditions would increase the female participation rate in the labor market.

Manual workers, who had a 3.3-fold higher risk of IHR in our analysis, usually need physical work capabilities, which tend to decline after the age of 30; this tendency can become critical in advanced years unless the physical demands of the work decline [19]. A prospective population-based study among middle-aged men suggested that physical workload increases the risk of retirement on a disability pension, particularly due to musculoskeletal disorders [20]. In heavy physical work, the risk of musculoskeletal disease increases in general and, among men with musculoskeletal disease, IHR experiences increase consequently.

IHR was frequent among the following, in order: those with psychiatric disease, ophthalmologic disease, neurologic disease, infectious disease, musculoskeletal disease, urologic disease, and circulatory disease (Table 1). The prevalence and incidence rates of those diseases increase greatly with age and disease severity. The development of those diseases can have deleterious effects on remaining in paid labor. These results are support Kang et al. [18], who suggested that workers with hypertension, diabetes mellitus, malignancy, cardiovascular disease, cerebrovascular disease, and arthritis were more likely to experience IHR.

We observed that specific diseases can lead to labor force exits (i.e., cardiovascular disease, cerebrovascular disease, malignancy, and musculoskeletal diseases). In a European study of 11,462 participants across ten European countries, the authors also found an association between being out of the labor force and chronic disease (e.g., depression, stroke, diabetes, and musculoskeletal diseases [21]).

Nonetheless, little reliable evidence exists regarding which specific diseases are major causes of IHR; thus, analysis of specific diseases leading to IHR may contribute to the development of strategies targeting specific disease. Our analysis shows that viral hepatitis, ischemic heart disease, congestive heart failure, cerebral stroke, alcoholism, insomnia, urolithiasis, pneumoconiosis, and vertigo strongly increase IHR risk (S1 Table). Part of the associations between specific diseases and IHR which we found in this study are also discussed in previous studies [18, 21]. In addition, we found that some diseases with high prevalence in Asia can be much heavier disease burden to IHR in Asian societies than Western countries (i.e., viral hepatitis, retinal disease et al). We also suggest that some disease can be severe IHR cause in relations with unhealthy habits (i.e., viral hepatitis and heavy alcohol drinking). Viral hepatitis is unique in Asian countries. Viral hepatitis infection has a high prevalence in East Asia and is a heavy health burden to society. In Western countries, viral hepatitis infection is comparatively rare and infects primarily in adulthood; in many Asian countries, chronic hepatitis B or C virus infections are common and often acquired perinatally or in childhood [22]. Viral hepatitis infection can lead to IHR with chronic hepatitis, liver cirrhosis, and hepatocellular carcinoma. Preventive management of viral hepatitis (e.g., via vaccination) may prevent some labor force exits.

Regarding ophthalmologic disease, cataract and retinal diseases, which are the main causes of blindness, had high hazard ratios (HR: 4.831, 7.427, respectively). Cataract and diabetic retinopathy are common vision-threatening diseases in the working-age population [23]. The global burden of blindness is an increasing problem. Whereas more blindness and visual impairment is found in developing countries, the economic burden is higher in developed countries (e.g., the US and Japan [24]). Labor force exits would decrease if vision-preserving management were made more affordable.

Unhealthy behavior (i.e., smoking, lack of regular exercise, obesity, heavy alcohol drinking, sleeping <6 hours per night) was also related to IHR in our sample. Such health related habits are associated with unemployment [21] [25].

Among these health related habits, alcoholism is a serious problem for IHR in Korea. It has been estimated that alcohol consumption costs 2.86% of Korea’s gross domestic product (GDP); this is much higher than in other industrialized countries (1.00–1.42% of GDP [26]). Moreover, heavy alcohol consumption and chronic viral hepatitis infection, which are common in Korea, have compounding effects on more serious disorders, such as liver cirrhosis or hepatocellular carcinoma (HCC) [27].

Poor sleep quality and limited total sleep may be associated with negative employment outcomes. In our study, adults who slept <6 hours and those who suffered from insomnia were more likely to report early labor force exit. Poor sleep quality and limited total sleep is associated with a range of medical conditions, accidental injuries, and work performance [28, 29]. Although, the prior studies did not investigate whether poor sleep quality and limited total sleep were associated with IHR, individuals who have medical conditions, experience accidental injuries, or have poor work performance may experience difficulty remaining in paid employment.

The effect of illness and unhealthy behavior on health-related early retirement in Korea was investigated in a study recently using the KLoSA database [18]; the present study goes a step further. First, our study included individuals aged 20–70 years, whereas the KLoSA study only included those aged >45 years. Therefore, we may generalize our results to most age groups and suggest that age predicts IHR (Table 3). Second, our study has greater statistical power for risk factor investigations, as it included 9,359 respondents, almost 3 times that of Kang et al. (3,371 adults). Third, our study analyzed job classification’s association with unwanted IHR, finding that service and sales workers and manual workers have a higher risk of IHR compared to managers and professionals. Fourth, we analyzed specific disease classifications leading to unwanted IHR, yielding findings representative of Asian endemic disease features. Finally, we calculated the lost years of working life expectancy according to the demographic and clinical characteristics of Korean adults, allowing us to compare each of the predictors affecting IHR.

This study has the following limitations. First, the analyzed data were collected over five years; this was insufficient to analyze long-term effects. Second, we could not take into account specific South Korean cultural aspects regarding welfare and employment security due to a lack of data. IHR will be a greater financial concern if employment security is not guaranteed, and it is necessary to take into account the influence of the Korea-specific social security system and distinguish between involuntary early retirement and voluntary retirement in further research. Third, it may seem obvious that individuals with disease and older individuals will retire due to ill health; however, we subdivided demographic characteristics, chronic disease, and unhealthy behaviors affecting IHR in South Korea and analyzed the HRs of those risk factors respectively to compare their effect on IHR. We aim to put our results to practical use to prioritize control measures for each risk factor to prevent IHR. It is therefore necessary to consider the purposes of this study in order to interpret the present results. Fourth, we used individuals without any disease as the reference group; therefore, the risk of IHR may be over-estimated for some specific diseases.

Despite these limitations, our results provide general insight regarding an aging society and related health and welfare issues, as well as analysis of a particular aging Asian population. Previous research has mainly addressed IHR in Europe, North America, and Australia, limiting the generalizability of available data to the Asian context in general and the South Korean context in particular. Moreover, our research identified a broad range of factors predicting IHR, including demographic and disease-specific features, whereas the concerns of previous studies were limited to associations between individuals’ health behavior and IHR. Finally, we used data from the KHPS, which is a national public database with non-institutionalized Korean adults populations 20 year old or more and collected data for a total of total of 7,201 households and 22,595 individuals, with a response rate of 94.1%.; therefore, the results reliably reflect the Korean population.

## Conclusion

The main purpose of this study was to identify demographic and clinical factors and unhealthy behaviors predicting IHR in the Korean population. We found that female, older, and manual workers were more likely to experience IHR. Moreover, IHR was more common among respondents with poor health-related habits (e.g., heavy drinking, irregular meals, fewer sleep hours, obesity, no regular exercise). Chronic diseases most closely associated with IHR were psychiatric disease, ophthalmologic disease, neurologic disease, infectious disease, and musculoskeletal disease. The average reduction in working life expectancy was 9.73 years. Most modern industrial societies are facing a decreasing rate of labor force participation and early retirement of older workers. IHR must therefore be regarded as a social issue relevant to public health and social welfare. We consider that our results may contribute to the development of strategies to reduce the risk of IHR and promote sustainable labor force participation in an aging society.

## Supporting Information

S1 Table Frequencies of ill health retirement and hazard ratios according to specific diseases. (DOCX)
