# Supplementary material for: Chronic Diseases, Health Behaviors, and Demographic Characteristics as Predictors of Ill Health Retirement: Findings from the Korea Health Panel Survey (2008–2012)
Source: PLoS One. 2016 Dec 8;11(12):e0166921. doi: 10.1371/journal.pone.0166921 (PMC5145165; doi:10.1371/journal.pone.0166921)
Supplement: S1 Table — (DOCX) [file pone.0166921.s001.docx]

| **Supplementary Table 1.** Frequencies of ill health retirement and hazard ratios according to specific diseases | | | | | | |
| --- | --- | --- | --- | --- | --- | --- |
|  | Total | Ill health retirement | | HR | 95% CI | |
|  |  | n | % |  |  |  |
| **Infectious disease** |  |  |  |  |  |  |
| Viral hepatitis | 70 | 11 | 15.71 | 13.929 | 6.809 | 28.493 |
| Mycosis | 140 | 14 | 1.27 | 5.141 | 2.726 | 9.694 |
| **Cancer** |  |  |  |  |  |  |
| Cancer of stomach | 16 | 3 | 18.75 | 8.857 | 2.661 | 29.477 |
| Cancer of bladder | 5 | 1 | 20 | 23.377 | 3.134 | 174.38 |
| Uterine myoma | 30 | 1 | 3.33 | 3.977 | 0.54 | 29.27 |
| **Hematologic disease** |  |  |  |  |  |  |
| Anaemia | 43 | 1 | 2.33 | 1.371 | 0.187 | 10.076 |
| **Endocrine disease** |  |  |  |  |  |  |
| Thyroid disorder | 83 | 3 | 3.61 | 0.856 | 0.117 | 6.263 |
| Diabetes mellitus | 338 | 3 | 8.88 | 4.08 | 2.469 | 6.744 |
| Malnutrition |  |  |  | 52.567 | 6.868 | 402.327 |
| Dyslipidaemia | 188 | 10 | 5.32 | 2.609 | 1.279 | 5.325 |
| **Psychiatric disease** |  |  |  |  |  |  |
| Alcoholism | 40 | 8 | 20 | 14.727 | 6.725 | 32.247 |
| Other behavioural disorders | 24 | 3 | 12.5 | 4.023 | 1.22 | 13.268 |
| **Neurologic disease** |  |  |  |  |  |  |
| Migraine | 59 | 4 | 6.78 | 3.811 | 1.343 | 10.817 |
| Insomnia | 22 | 6 | 27.27 | 22.235 | 9.002 | 54.918 |
| **Ophthalmologic disease** |  |  |  |  |  |  |
| Conjunctivitis | 12 | 1 | 8.33 | 6.835 | 0.925 | 50.503 |
| Cataract | 65 | 10 | 15.38 | 4.831 | 2.276 | 10.254 |
| Retina disease | 28 | 9 | 32.14 | 7.427 | 3.355 | 16.439 |
| Dysopsia | 111 | 9 | 8.11 | 5.828 | 2.793 | 12.162 |
| **Otologic disease** |  |  |  |  |  |  |
| Otitis media | 33 | 3 | 9.09 | 2.262 | 0.308 | 16.603 |
| **Circulatory disease** |  |  |  |  |  |  |
| Hypertension | 930 | 47 | 5.05 | 1.967 | 1.246 | 3.103 |
| Ischemic heart disease | 47 | 7 | 14.89 | 13.369 | 5.863 | 30.487 |
| Dysrhythmia | 13 | 2 | 15.38 | 8.426 | 2.007 | 35.373 |
| Congestive heart failure | 12 | 2 | 15.38 | 32.578 | 12.967 | 81.848 |
| Cerebral haemorrhage | 16 | 4 | 25 | 12.201 | 4.194 | 35.498 |
| Cerebral infarction | 26 | 6 | 23.08 | 12.953 | 5.366 | 31.265 |
| Haemorrhoid | 67 | 1 | 1.49 | 0.827 | 0.113 | 6.07 |
| **Respiratory disease** |  |  |  |  |  |  |
| Chronic sinusitis | 21 | 4 | 19.05 | 9.306 | 3.215 | 26.934 |
| Rhinitis | 272 | 2 | 0.74 | 0.842 | 0.203 | 3.491 |
| Chronic obstructive pulmonary disease | 17 | 1 | 5.88 | 2.439 | 0.332 | 17.932 |
| Pneumoconiosis | 4 | 1 | 25 | 11.252 | 1.496 | 84.617 |
| **Digestive disease** |  |  |  |  |  |  |
| Dental caries | 170 | 7 | 4.12 | 2.27 | 1.004 | 5.133 |
| Gingivitis | 311 | 25 | 8.04 | 3.917 | 2.296 | 6.684 |
| Peptic ulcer | 49 | 1 | 2.04 | 1.241 | 0.169 | 9.091 |
| Gastritis and duodenitis | 285 | 23 | 8.07 | 2.626 | 1.412 | 4.884 |
| Gastroesophageal reflux disease | 123 | 15 | 12.2 | 5.674 | 2.972 | 10.832 |
| Functional dyspepsia | 80 | 2 | 2.5 | 1.778 | 0.427 | 7.4 |
| Fatty liver | 103 | 3 | 2.91 | 2.474 | 0.761 | 8.045 |
| **Musculoskeletal disease** |  |  |  |  |  |  |
| Rheumatoid arthritis | 85 | 3 | 3.53 | 2.168 | 0.665 | 7.065 |
| Osteoarthritis | 414 | 37 | 8.94 | 3.021 | 1.841 | 4.958 |
| Herniation of intervertebral disc | 174 | 6 | 3.45 | 2.371 | 0.996 | 5.643 |
| Non-specific back pain | 82 | 8 | 9.76 | 4.22 | 2.475 | 7.197 |
| Myofascial pain syndrome | 255 | 8 | 3.14 | 1.661 | 0.697 | 3.96 |
| Osteopenia and osteoporosis | 82 | 8 | 9.76 | 2.634 | 1.089 | 6.367 |
| **Genitourinary disease** |  |  |  |  |  |  |
| Renal failure | 13 | 7 | 53.85 | 38.69 | 16.883 | 88.662 |
| Urolithiasis | 18 | 1 | 5.56 | 12.435 | 1.685 | 91.737 |
| Postmenopausal syndrome | 52 | 1 | 1.93 | 1.436 | 0.195 | 10.569 |
| **Others** |  |  |  |  |  |  |
| Dizziness and vertigo | 18 | 6 | 33.33 | 9.734 | 3.88 | 24.421 |
| Chronic fatigue | 17 | 2 | 11.76 | 5.024 | 1.173 | 21.516 |
